# IL-33 Effect on Quantitative Changes of CD4^+^CD25^high^FOXP3^+^ Regulatory T Cells in Children with Type 1 Diabetes

**DOI:** 10.1155/2016/9429760

**Published:** 2016-09-28

**Authors:** Monika Ryba-Stanisławowska, Paulina Werner, Maria Skrzypkowska, Agnieszka Brandt, Jolanta Myśliwska

**Affiliations:** ^1^Department of Immunology, Medical University of Gdańsk, Dębinki 1, 80-211 Gdańsk, Poland; ^2^Clinic of Pediatrics, Department of Diabetology and Endocrinology, Medical University of Gdańsk, 80-211 Gdańsk, Poland

## Abstract

IL-33 is an IL-1 cytokine family member, with ability to induce both Th1 and Th2 immune responses. It binds to ST2 receptor, whose deficiency is associated with enhanced inflammatory response. The most recent studies have shown the immunoregulatory effect of IL-33 on Tregs in animal models. As type 1 diabetes is an autoimmune, inflammatory disease, where Treg defects have been described, we aimed to analyze the* in vitro* influence of recombinant IL-33 on quantitative properties of regulatory CD4^+^CD25^high^FOXP3^+^ T cells. CD4^+^CD25^high^FOXP3^+^ as well as CD4^+^CD25^high^FOXP3^+^ST2^+^ Tregs were analyzed by flow cytometry. In a group of patients with type 1 diabetes* in vitro* IL-33 treatment induced regulatory CD4^+^CD25^high^FOXP3^+^ cell frequencies as well as upregulating the surface expression of ST2 molecule. In addition, the number of CD4^+^CD25^high^FOXP3^+^ cells carrying ST2 receptor increased significantly. Similar effect was observed in case of the FOXP3 expression. We did not observe any significant changes in IL-33 treated cells of healthy controls. The level of ST2 was higher in serum of patients with type 1 diabetes in comparison to their healthy counterparts. We propose that IL-33 becomes an additional immunostimulatory factor used to induce Treg expansion in future clinical trials of adoptive therapy in type 1 diabetes.

## 1. Introduction

IL-33 is a member of the IL-1 cytokine family that was identified in 2005 [1]. In contrast to other IL-1 family members, IL-33 was mostly described as Th2 type response-inducing cytokine [1–3]. It binds to the ST2 receptor that is expressed on various cells of the immune system, such as Th2 lymphocytes, mast cells, basophils, or eosinophils [1, 2, 4], but not Th1 cells [5]. However, Th1 immune response can be also initiated by IL-33 [6]. IL-33 alone or in synergy with IL-12 upregulates IFN-*γ* expression in NK or NKT cells [6, 7], which in turn promotes the activity of Th1 lymphocytes. So, despite the lack of ST2 receptor, IL-33 may have an indirect effect on the induction of Th1 immune response. ST2 receptor can be present in two forms—soluble and membrane-bound [1]. It was suggested that ST2 deficiency alters Th1/Th2 balance and leads to enhanced inflammatory response [8, 9]. In addition, it was shown that deletion of* ST2* gene is accompanied by greater susceptibility to T cell mediated organ-specific autoimmune diseases [10]. BALB/c mice treated with anti-ST2 antibodies showed weakened Th2 immunity and overactive Th1 response [11].

The role of IL-33 in different diseases is dual and depends on the immune mechanism underlying the pathogenesis of each condition [12]. In mice model of type 2 diabetes (ob/ob) or Crohn's disease, conditions which are characterized by profound Th1 immune response, IL-33 induced Th2 cells and thus protected against inflammation [13, 14]. However, in mice model of ulcerative colitis, whose pathophysiology is linked to Th2 cytokines, the harmful effect of IL-33 was seen, despite its ability to inhibit Th1-related cytokines in these mice [15].

The overactive Th1 immune response is controlled by regulatory T cells (Tregs), which play a crucial role in maintaining immune homeostasis. However their quantitative and/or qualitative defects are frequently observed in several autoimmune and/or inflammatory diseases including type 1 diabetes (DM1) [16, 17]. Therefore, new biological agents need to be analyzed with respect to their ability to increase Treg number and function.

The only published data on the role of IL-33 on regulatory T cells come from studies on animal models [14, 18, 19]. The use of IL-33 in those studies showed promising results in promoting regulatory phenotype [14, 18, 19]. Type 1 diabetes is characterized by the enhanced inflammatory response as well as regulatory T cell deficiency [16, 20]. Moreover, there are no data regarding the influence of IL-33 on regulatory T cell subset in DM1 patients. Taking into account these facts, the aim of the current study was to analyze the* in vitro* effect of IL-33 on the quantitative properties of regulatory CD4^+^CD25^high^FOXP3^+^ T cell population in patients with type 1 diabetes.

## 2. Material and Methods 

### 2.1. Study Groups

The study group consisted of 16 young patients recruited from the Clinic of Pediatrics, Department of Diabetology and Endocrinology, Medical University of Gdańsk, with diagnosed type 1 diabetes and 8 healthy individuals treated as a control group. The diagnosis of type 1 diabetes was made in accordance with the American Diabetes Association criteria [21]. At the time of sampling, a blood glucose level along with biochemical measurement of renal function, CRP, and glycosylated hemoglobin (HbA1c) was taken. All DM1 patients enrolled in the study were free of microvascular complications and any other autoimmune, chronic, and acute inflammatory diseases. Clinical characteristics of DM1 patients are presented in Table 1. The control group consisted of age and sex matched healthy individuals recruited during control visits in outpatient clinic. No signs of autoimmune, chronic, inflammatory, or neoplastic disease at the time of sampling and no evidence of DM1 in their families were disclosed as confirmed by medical records, laboratory examination, and laboratory tests. The study followed the principles of the Declaration of Helsinki and was approved by the Ethics Committee of the Medical University of Gdańsk.

### 2.2. Cell Isolation and Culture

Heparin-stabilized venous blood samples were collected aseptically and used to isolate peripheral blood mononuclear cells (PBMCs) by Histopaque-1083 (Sigma) density gradient centrifugation. Cells were suspended at a density of 1 × 10^6^ cells/mL and cultured in 1 mL RPMI 1640 supplemented with 5% heat-inactivated fetal calf serum (FCS). For each study individual, the following pattern of stimulation was used: 1st control well designated as “nonstimulated” and 2nd well designated as “stimulated.” Cells in both wells were treated with anti-CD3 (clone UCHT1, BioLegend, USA) and anti-CD28 (clone CD28.2, BioLegend, USA) antibodies, both at 5 *μ*g/mL. In addition, recombinant IL-33 (BioLegend, USA) at a concentration 25 ng/mL was added to wells designated as “stimulated.” Cell cultures were incubated at 37°C with 5% CO_2_ for 24 h.

### 2.3. Flow Cytometric Staining and Analysis

Cells were harvested, washed with Cell Staining Buffer (BioLegend, USA), and stained with antibodies for Treg analysis: anti-CD4 (IgG1, mouse APC, Clone RPA-T4, BioLegend, USA), anti-CD127 (IgG1, mouse PE-Cy7, Clone A019D5, BioLegend, USA), anti-CD25 (IgG1, mouse PerCP-Cy5.5, Clone BC96, BioLegend, USA), and anti-ST2 (IgG1 mouse PE, Clone 97203, R&D, USA) antibodies. After 20 minutes of incubation at room temperature, cells were washed and stained for intracellular expression of FOXP3 (IgG1, mouse FITC, Clone 206D, BioLegend, USA). Intracellular staining for FOXP3 was performed according to the manufacturer's suggestions (BioLegend, USA). Expression of cell surface and intracellular markers was assessed using flow cytometry (LSRII, Becton Dickinson, USA) after gating on live cells determined by scatter characteristics. Positive signal for each stain was established using appropriate isotype controls. In addition, the expression of FOXP3 and ST2 in the CD4^+^CD25^high^FOXP3^+^ gate was quantified by determining Mean Fluorescence Intensity (MFI). It was quantified as a ratio of Mean Fluorescence Intensity for FOXP3/ST2 to MFI for appropriate isotype control. Data were analyzed by FACSDiva 6.0 software (Becton Dickinson, USA).

### 2.4. Determination of Serum ST2 Level

Serum level of ST2 (sST2) was measured by ELISA method (Quantikine R&D Systems, Minneapolis, MN, USA) according to the manufacturer protocol. Minimum detectable concentration was determined by the manufacturer as 5.1 pg/mL. The results were read on the automated plate reader (Multiscan MCC/340, Labsystems, Helsinki, Finland).

### 2.5. Statistical Analysis

The results were analyzed using Statistica, ver. 10.0 (StatSoft Inc., USA). For comparison of the skew-distributed variables, nonparametric Mann–Whitney *U* or Wilcoxon tests were applied. Spearman's correlations were used to compare the associations between analyzed parameters. The level of significance was set at *p* ≤ 0.05.

## 3. Results

**Figure 1:**
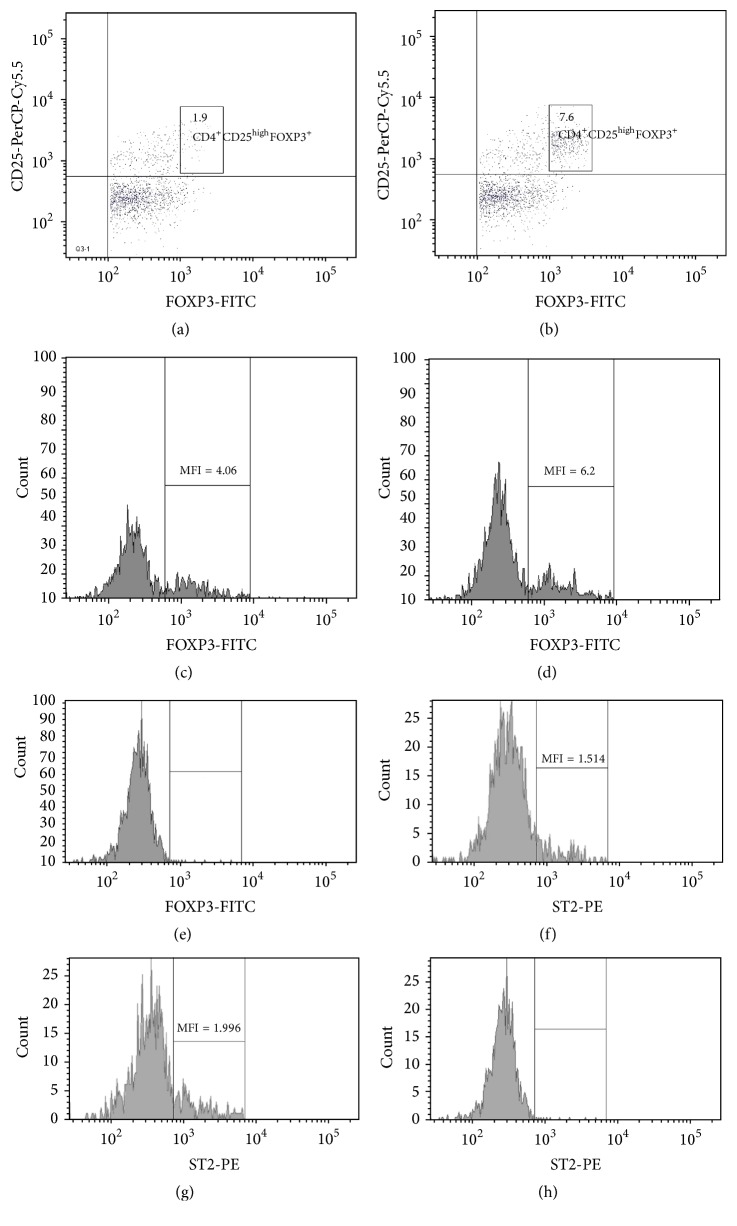
Representative flow cytometric analysis of CD4^+^CD25^high^FOXP3^+^ T cells in DM1 patient after* in vitro* IL-33 treatment. PBMCs from diabetic type 1 patient were cultured and stimulated as described in Material and Methods, stained with antibodies against Treg associated molecules, and analyzed using flow cytometry. Analyzing CD4^+^CD25^high^FOXP3^+^ T cells, dot plots representing anti-CD4 versus SS were carried out to establish CD4^+^ and CD4^−^ lymphocyte gates. Then, the following dot plots were generated: anti-CD4 versus CD127 from CD4^+^ gate. After gating on CD4^+^127^−^ cells, the frequency of nonstimulated (a) and IL-33 stimulated (b) CD4^+^CD25^high^FOXP3^+^ cells was determined. Gated, nonstimulated (c) and IL-33 stimulated (d) CD4^+^CD25^high^FOXP3^+^ T cells were checked for the expression of FOXP3 defined as Mean Fluorescence Intensity. Similarly, the expression of ST2 on nonstimulated (f) and IL-33 stimulated (g) CD4^+^CD25^high^FOXP3^+^ was analyzed. During the analysis isotypes controls for FOXP3 (e) and ST2 (h) staining were used. MFI: Mean Fluorescence Intensity.

**Figure 2:**
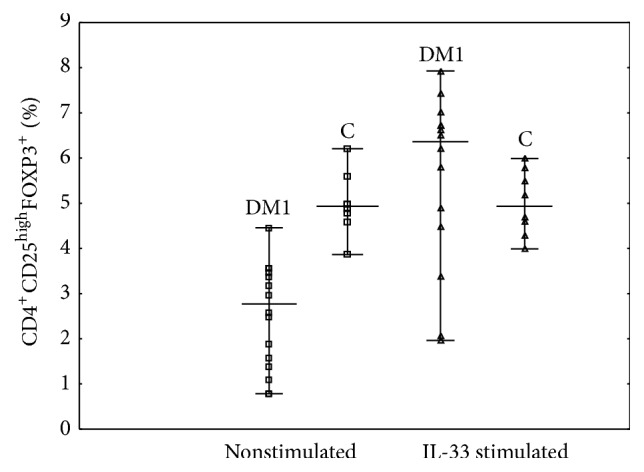
Quantitative characteristics of CD4^+^CD25^high^FOXP3^+^ Tregs after* in vitro* treatment with IL-33 in DM1 patients and control group. PBMCs from 16 patients with type 1 diabetes and 8 healthy individuals were stimulated as described in Material and Methods and stained with specific antibodies against Treg associated molecules. During the cytometric analysis, the frequency of CD4^+^CD25^high^FOXP3^+^ was determined. Data were calculated with the nonparametric Wilcoxon test. Horizontal lines represent the mean frequency of cells. DM1: group of patients with type 1 diabetes; C: control group of healthy individuals.

In a group of patients with type 1 diabetes* in vitro* IL-33 treatment induced regulatory CD4^+^CD25^high^FOXP3^+^ cells (Figures 1 and 2). After stimulation with IL-33, the frequency of CD4^+^CD25^high^FOXP3^+^ has increased significantly (Table 2; *p* = 0.0004). This was in contrast to the control group, which showed no significant changes in Treg frequencies after IL-33 treatment (Figure 2; Table 3). In addition, we have noticed that* in vitro* IL-33 treatment significantly induced the frequency of Tregs expressing ST2 molecule, which was seen only in type 1 diabetic group (Table 2). What is more, the expression of FOXP3 transcription factor defined as Mean Fluorescence Intensity (MFI) was higher in IL-33 stimulated cells in comparison to their nonstimulated counterparts (Figures 1(c) and 1(d); Table 2). Similar results were obtained in case of ST2 expression. It was upregulated on cells from patients with type 1 diabetes cultured in medium containing IL-33 (Figures 1(f) and 1(g); Table 2). In a healthy control group, Tregs showed some level of ST2 expression, which however did not change significantly after IL-33 treatment (Table 3).

**Figure 3:**
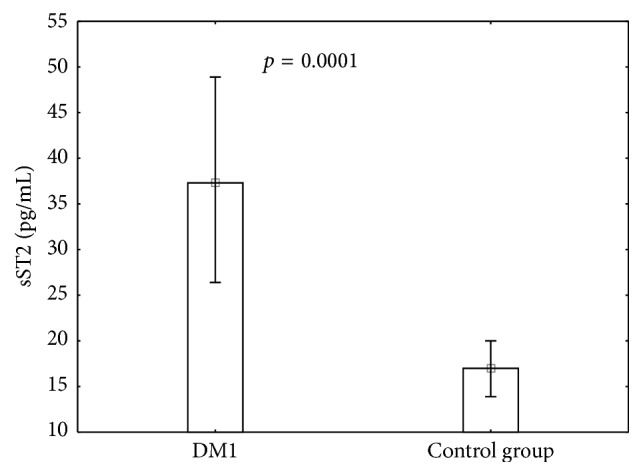
Serum ST2 level in patients with type 1 diabetes and healthy individuals from the control group. The level of serum ST2 (sST2) was measured by ELISA in all study individuals. The mean value (10/90 percentiles) of sST2 was 37.3 (26.4/48.9) and 17 (13.9/20) pg/mL for DM1 and control group, respectively. Differences were calculated by the Mann–Whitney *U* test.

To extend our study, we analyzed the concentration of soluble ST2 in serum of all individuals enrolled in the study. We found that ST2 level was significantly higher in patients with type 1 diabetes compared with healthy subjects (Figure 3; *p* = 0.0001). Moreover, type 1 diabetic patients with higher frequency of ST2 expressing CD4^+^CD25^high^FOXP3^+^ T cells had lower serum ST2 level (*r* = [−0.25]; Pearson correlation). Analysis of an association between FOXP3 and ST2 expression in IL-33 stimulated and nonstimulated cell cultures showed that in both cases FOXP3 expression was positively correlated with the expression of ST2 molecule (Figure 3).

## 4. Discussion

To date, there are no data on the influence of IL-33 on FOXP3^+^ regulatory T cells in patients with type 1 diabetes. There are only few studies which showed IL-33 immunoregulatory effect on this cell subset in mouse model of inflammatory bowel disease [14] and in mice after heart transplantation [19]. In both of these studies, IL-33 was shown to upregulate CD4^+^FOXP3^+^ Tregs by increasing their numbers, enhancing suppressive activity, as well as ST2 surface expression. In addition, two recently published papers have confirmed that IL-33 has the ability to induce regulatory phenotype by promoting the expansion of ST2^+^ Tregs [19, 22].

**Figure 4:**
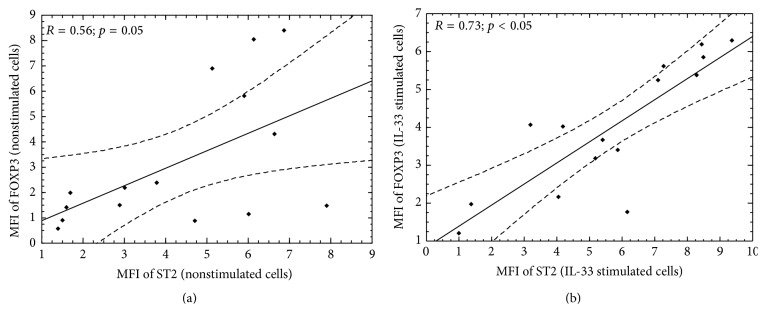
Association between the expression of FOXP3 and that of ST2 among CD4^+^CD25^high^FOXP3^+^ cells after IL-33* in vitro* treatment in DM1 patients. Expression values of FOXP3 and ST2 defined as MFI among nonstimulated (a) and IL-33 stimulated (b) CD4^+^CD25^high^FOXP3^+^ cells in a group of patients with type 1 diabetes were correlated. The Spearman test was used to calculate the strength of correlation. MFI: Mean Fluorescence Intensity.

Surface ST2 expression can be found on Th2 lymphocytes, as well as Tregs, though to a lesser extent, but not Th1 cells [5, 18, 22, 23]. It depends on STAT5 and GATA3 nuclear activation [24]. Interestingly, regulatory T cells also require STAT5 as well as GATA3 for their proper function [25, 26]. GATA3^−/−^ Tregs have been shown to be defective in their suppressive function. They downregulated FOXP3 expression and were more susceptible to Th17 inflammatory phenotype transformation [27]. One may suspect that if Tregs have impairments in STAT5/GATA3 signaling pathway, the reduced ST2 expression may attenuate their suppressive activity [18]. All the more so because ST2^+^ Tregs represent an activated subset of FOXP3^+^ cells with high suppressive activity [19]. In the current study, we found low level of ST2 expression on Tregs treated only with anti-CD3/anti-CD28 antibodies, which confirms available published data that Tregs express ST2 receptor.* In vitro* IL-33 treatment however increased the frequency of CD4^+^CD25^high^FOXP3^+^ cells expressing ST2 molecule as well as its surface expression. These effects were not observed in case of cells from healthy subjects. Moreover, the expression of ST2 increased along with the expression of FOXP3, which suggests that genes encoding both of these molecules might depend on each other. ST2^+^FOXP3^+^ Tregs have been shown to express high levels of GATA3 transcription factor [22] which is activated exactly by IL-33 [23]. What is more, GATA3 is also activated by IL-2 [25]. IL-2 deficient mice do not express GATA3 and have reduced Treg frequencies [25]. Studies by Matta et al. showed that IL-33 stimulated dendritic cells secrete IL-2 to selectively expand ST2^+^ suppressive Tregs [19]. IL-2 increased ST2 expression on both dendritic cells and interacting Tregs [19], which may be due to activation of GATA3 transcription factor. It seems then that levels of ST2 and FOXP3 expression are tightly related, and such correlation was shown by us (Figure 4). In agreement with previous research [18, 19], our study indicates that IL-33 upregulates ST2 expression on Treg cells, inducing at the same time FOXP3 transcription factor. Based on our study results and other available data, we can suspect that expression of FOXP3 along with ST2 may reflect Treg activity level, and this should be analyzed in more detail in future experiments. Certainly, this is a right direction for additional research to further explore the association between ST2/IL-33 axis and Tregs.

It was shown that patients with autoimmune diseases, as well as those with type 2 diabetes, have elevated levels of soluble form of ST2 [28–30]. Here we have confirmed such an association. Patients with type 1 diabetes had higher level of serum ST2 in comparison to healthy individuals. Moreover, the lower the ST2^+^ Tregs frequency was, the more the sST2 in a serum was observed. This may suggest that in inflammatory microenvironment ST2-positive cells shed this molecule from the cell surface and thus lose their phenotypical and/or functional properties. This is more likely in view of the studies, which showed that deletion of* ST2* gene in BALB/c mice leads to enhanced susceptibility to streptozotocin-induced diabetes [8, 9]. Moreover, the diabetic* ST* ^−/−^ mice produced elevated levels of proinflammatory cytokines [8, 9]. Proinflammatory cytokines enhance the secretion of sST2, which was shown both* in vitro* and* in vivo* [31].

Based on the available scientific data, one cannot say with certainty that IL-33 has pro- or anti-inflammatory activity; however one can suspect that its action may depend on the cytokine microenvironment responsible for the disease pathology. In our previous study, we have reported that inflammatory environment might be responsible for Treg quantitative and qualitative defects [32]. We found that treatment of Tregs with anti-TNF antibody restored their impaired phenotype and induced the frequency of FOXP3 expressing cells. Here, we propose an additional factor to be examined that may induce regulatory T cells and thus become new immunotherapeutic tool for the adoptive therapies of type 1 diabetes, as well as other autoimmune and inflammation-related diseases that are characterized by defects in Treg-mediated regulation. IL-33 could induce Treg cells in patients with type 1 diabetes; however, more studies including qualitative characterization of IL-33 treated CD4^+^CD25^high^FOXP3^+^ on a larger group of patients need to be done. It would help further understand the relationship between Tregs and ST2/IL-33 axis.

To our knowledge, this is the first study that suggests the association between ST2/IL-33 axis and regulatory T cells in human type 1 diabetes.

## Figures and Tables

**Table 1:** Clinical characteristics of patients with type 1 diabetes.

Clinical parameter	Mean ± standard deviation
Age (years)	11.2 ± 3.3
Duration of diabetes (years)	5 ± 2.7
BMI (kg/m^2^)	19.6 ± 3.67
CRP (mg/L)	4.09 ± 1.6
HbA1c (%)	8.5 ± 1.4
Serum creatinine level (mg/dL)	0.62 ± 0.13
Serum albumin level (g/L)	22.32 ± 18.15

Data are shown as mean ± standard deviation.

**Table 2:** The status of CD4^+^CD25^high^FOXP3^+^ and CD4^+^CD25^high^FOXP3^+^ST2^+^ Treg cells after *in vitro* treatment with IL-33 in patients with type 1 diabetes.

	CD4^+^CD25^high^FOXP3^+^ (%)	CD4^+^CD25^high^FOXP3^+^ST2^+^ (%)	MFI of FOXP3 among CD4^+^CD25^high^FOXP3^+^ cells	MFI of ST2 among CD4^+^CD25^high^FOXP3^+^ cells
Nonstimulated	2.8 (0.8/4.7)	0.85 (0.4/1.8)	2.1 (0.8/5.81)	4.92 (1.5/6.87)
IL-33 stimulated	6.35 (2.0/8.1)	2.05 (0.6/4.1)	4.06 (1.98/7)	5.54 (3.19/8.49)
*p*	0.0004	0.0019	0.007	0.02

MFI: Mean Fluorescence Intensity.

Differences were calculated with the nonparametric Wilcoxon test.

Data are presented as median and 10th/90th percentile.

**Table 3:** The status of CD4^+^CD25^high^FOXP3^+^ and CD4^+^CD25^high^FOXP3^+^ST2^+^ Treg cells after *in vitro* treatment with IL-33 in control group.

	CD4^+^CD25^high^FOXP3^+^ (%)	CD4^+^CD25^high^FOXP3^+^ST2^+^ (%)	MFI of FOXP3 among CD4^+^CD25^high^FOXP3^+^ cells	MFI of ST2 among CD4^+^CD25^high^FOXP3^+^ cells
Nonstimulated	4.9 (3.7/6.8)	2.5 (1.4/3.0)	4.12 (1.4/7.3)	6.3 (1.6/8.22)
IL-33 stimulated	4.95 (4.0/6.0)	2.55 (0.6/3.6)	4.15 (1.2/5.2)	5.63 (0.99/8.0)
*p*	0.57	0.75	0.34	0.22

MFI: Mean Fluorescence Intensity.

Differences were calculated with the nonparametric Wilcoxon test.

Data are presented as median and 10th/90th percentile.
